# Near-infrared hyperspectral imaging for online measurement of the viability detection of naturally aged watermelon seeds

**DOI:** 10.3389/fpls.2022.986754

**Published:** 2022-11-07

**Authors:** Jannat Yasmin, Mohammed Raju Ahmed, Collins Wakholi, Santosh Lohumi, Perez Mukasa, Geonwoo Kim, Juntae Kim, Hoonsoo Lee, Byoung-Kwan Cho

**Affiliations:** ^1^ Department of Biosystems Machinery Engineering, College of Agricultural and Life Science, Chungnam National University, Daejeon, South Korea; ^2^ Department of Bio-Industrial Machinery Engineering, College of Agriculture and Life Science, Gyeongsang National University, Jinju-si, Gyeongsangnam-do, South Korea; ^3^ Institute of Smart Farm, Gyeongsang National University, Jinju-si, Gyeongsangnam-do, South Korea; ^4^ Department of Biosystems Engineering, College of Agriculture, Life & Environment Science, Chungbuk National University, Cheongju, Chungbuk, South Korea; ^5^ Department of Smart Agricultural Systems, College of Agricultural and Life Science, Chungnam National University, Daejeon, South Korea

**Keywords:** seed, viability, NIR imaging, online measurement, graphical interface

## Abstract

The viability status of seeds before sowing is important to farmers as it allows them to make yield predictions. Monitoring the seed quality in a rapid and nondestructive manner may create a perfect solution, especially for industrial sorting applications. However, current offline laboratory-based strategies employed for the monitoring of seed viability are time-consuming and thus cannot satisfy industrial needs where there is a substantial number of seeds to be analyzed. In this study, we describe a prototype online near-infrared (NIR) hyperspectral imaging system that can be used for the rapid detection of seed viability. A wavelength range of 900–1700 nm was employed to obtain spectral images of three different varieties of naturally aged watermelon seed samples. The partial least square discriminant analysis (PLS-DA) model was employed for real-time viability prediction for seed samples moving through a conveyor unit at a speed of 49 mm/sec. A suction unit was further incorporated to develop the online system and it was programmatically controlled to separate the detected viable seeds from nonviable ones. For an external validation sample set showed classification accuracy levels of 91.8%, 80.7%, and 77.8% in relation to viability for the three varieties of watermelon seed with healthy seedling growth. The regression coefficients of the classification model distinguished some chemical differences in viable and nonviable seed which was verified by the chromatographic analysis after the detection of the proposed online system. The results demonstrated that the developed online system with the viability prediction model has the potential to be used in the seed industry for the quality monitoring of seeds.

## Introduction

Watermelon (*Citrullus lanatus*) is one of the most economically important crops grown in 122 countries, in terms of production value (US$ 51 million in 2020) (Food and Agriculture Organization of the United Nations, 2022). The use of high-quality watermelon seeds is one of the most important elements in farming systems for increasing agricultural production (Ahmed et al., 2020). Each seed has its own distinct capability to produce a plant from a seed lot in the field under favorable conditions. From a technological point of view, a seed can be defined as a small embryonic plant with stored food enclosed within a cover or seed coat. The seed viability denotes the degree of metabolically active and alive seeds that can undergo the metabolic reactions required for germination and seedling growth. Determination of the seed quality is also critical for producers who need to predict seed viability using an appropriate methodology in a precise fashion (Mukasa et al., 2022).

To acquire improved cultivars, watermelon breeders need to have high-quality seeds of cultivars, breeding lines, and gene mutants that exhibit desired traits. To accomplish this, they should evaluate the seed samples of available cultivars to become familiar with germplasm diversity (Whitfield and Last, 2017). Therefore, the quick indication of seed viability has many advantages for watermelon breeders that can allow rapid decision making.

To determine the seed viability, the most used method is the germination rate (%), which has become universally accepted. Several renowned methods are also available for measuring the germination rate, such as the tetrazolium test (TZ), biochemical tests (coloring, enzyme activity, and oxidase methods); and other seed chemical tests (fatty acid, ferric chloride, and conductivity tests) (Copeland and McDonald, 2012). However, in general, seed industries detect nonviable seed by randomly taken of a handful seeds from a seed lot and perform the conventional germination test (Mukasa et al., 2022). Therefore, these germination test results are often not exact and sometimes may overestimate the field performance of a particular seed lot. In addition, the germination test requires a longer duration to ensure the seed quality as germination time varies depending on the seed species.

To overcome this weakness, seed industries need to undergo a continuous detection process, which should be an uninterrupted process that analyzes and controls the seed quality during the process. Continuous quality control requires the development of new technologies for real-time (online, in-line, or at-line) monitoring that can improve the quality, safety, and efficiency of seed quality detection. By combining spectroscopic technology with an imaging camera, manufacturers can continuously monitor each seed of a seed lot to predict the viability.

The hyperspectral (HSI) based sorting system has the great potential to replace the conventional germination testing techniques. By employing HSI technique, several important aspects of the seeds can be obtained. Published researches has already suggested the use of HSI incorporated with multivariate data analysis to predict different seed factors, such as seed age (Huang et al., 2016), viability detection (Ambrose et al., 2016), moisture content (Cogdill et al., 2004), and the detection of fungal infestation (Del Fiore et al., 2010; Kandpal et al., 2014). For actual real-time application, it is necessary to simultaneously collect and analyze data during the continuous movement of seeds. Previous researchers were able to apply the HSI technique for quality evaluation of the corn seed viability during continuous operation (Wakholi et al., 2018).

Watermelon seed both seeded and seedless contains many chemical and mineral components, and published literature has described the contents of carbohydrates, proteins, and moisture in different varieties (Jyothi lakshmi & Kaul, 2011; Tabiri, 2016). Researchers had reported that the loss of seed-soluble carbohydrates is related to seed aging (Horbowicz and Obendorf, 1994), and this has been proved for maize seed (Bernal-Lugo and Carl Leopold, 1992) and wheat grain seed (Lehner et al., 2008). Other published research has also confirmed that seed proteins (Mbofung et al., 2013; Sekar et al., 2015) and moisture content (Repo et al., 2002; Kibinza et al., 2006) have direct relationships with the seed viability. Research proved that the deficiency in the moisture content of a substance affects the physical and chemical properties of a material (Adebowale et al., 2011; Sun et al., 2017).

During aging process of seeds, their vitality steadily decrease in storage period. However, almost all above studies only focused on artificial seed aging. Moreover, previous published research has proved that artificially aged seeds display amplified deterioration in comparison with naturally aged seeds (Petruzzelli and Carella, 1983; Giurizatto et al., 2012; Fantazzini et al., 2018) and are associated with a higher prediction accuracy. In addition, all these researches are destructive, and limited research has been conducted for naturally aged seed deterioration. Hence, this research focuses on natural aging as researchers detected physiological changes in seeds after long term storage (Ahmed et al., 2018) that reduced the viability (Yasmin et al., 2019).

To the best of our knowledge, it was hard to find studies about high-throughput phenotyping techniques for watermelon seed quality focused on watermelon breeding issues. In our previous study, to distinguish viable and non-viable triploid watermelon seeds from three different varieties stored for four years, a machine learning based classification method was developed using Fourier transform near-infrared spectroscopy (Yasmin et al., 2019). On the other hand, only a few studies have been recently conducted to develop phenotyping technologies for the rapid and non-destructive prediction of watermelon seed qualities such as bacterial infection (Lee et al., 2017), detection of seedless from seeded watermelon seeds (Mukasa et al., 2022), and internal parameters (endosperm and air space) (Ahmed et al., 2020), and morphological features (Liu et al., 2019).

Therefore, this study proposes an online HSI system for the rapid and continuous identification of viable seed after natural aging. Moreover, this prototype system can be easily set up on the inspection lines to monitor, detect, and sort viable seeds from naturally aged seedless watermelon seed varieties. By applying this method, seed quality measurement can be performed for the entire batch in fast and cost-effective manner as the production cost of seedless watermelon seed is higher than the seeded watermelon seed (Seeds, 2014). To develop classification (viability of seeds) model, partial least square discriminant analysis (PLS-DA) technique and various was utilized and eight various preprocessing methods were applied for more accurate analysis. For viability prediction of watermelon seeds, pixel-based chemical imaging was performed for viability prediction of watermelon seeds by PLS-DA model. This could provide a hands-on solution for large-scale, real-time viability categorization of naturally aged seeds.

The aim of this study was to develop a prototype of an online NIR hyperspectral imaging system that can be used as a continuous detection process to visualize the viability of naturally aged seeds. To achieve this, a conveyor belt system was designed and equipped with an NIR-HSI camera, which was controlled by a custom-built graphical interface. By using this proposed system, real-time quality monitoring was used to assess the viability of bulk seed samples. To evaluate the detection accuracy of the proposed real-time NIR-HSI technique, a separate validation data set was used. After viable and nonviable seeds were detected as two groups, another germination test was performed to verify the germination accuracy. Afterwards, chromatographic and moisture content tests were conducted to identify designated chemical and moisture differences between viable and nonviable seed.

## Materials and methods

### Seed collection

**Figure 1:**
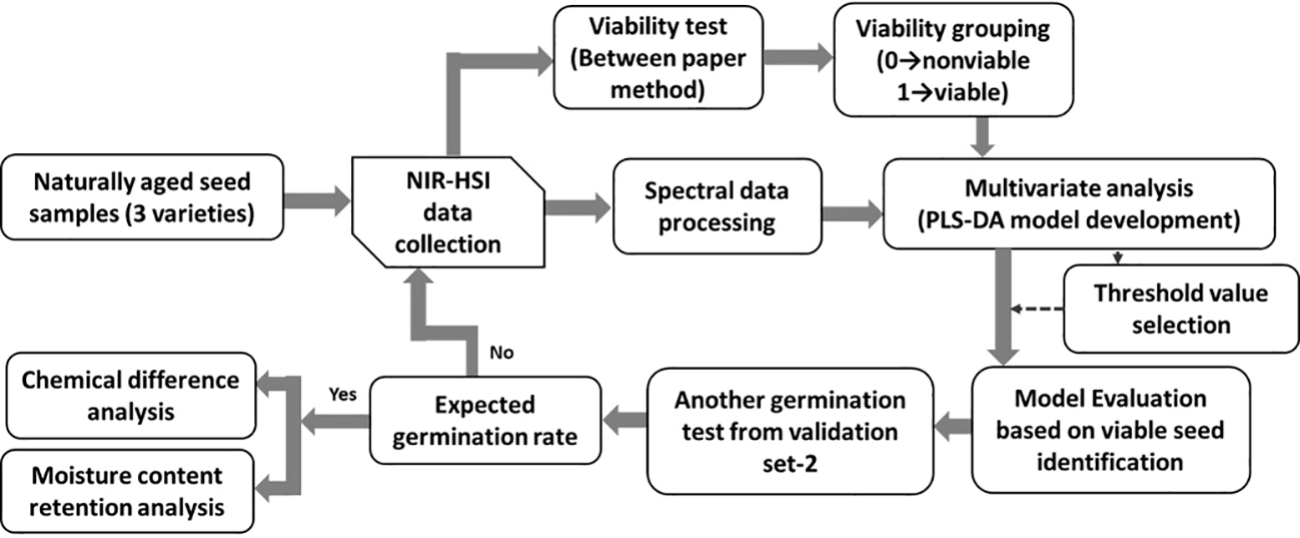
Procedure used to generate the classification model using the near infrared hyperspectroscopic (NIR-HSI) technique.

The three varieties of naturally aged seedless (triploid) watermelon seed used in this study, Choiganggul, Sambaechea, and Leehyunglim varieties, which will be referred to as V1, V2, and V3, respectively, were provided by the Korea seed & variety service (KSVS), South Korea. The seed samples were stored in a small room specifically designed for seed lots under the management of KSVS from 2015 for natural aging. Seeds were sealed in a plastic container, and the room temperature was controlled at around 5°C. The germination rate of the seed varieties was provided by the seed institute; 89%, 32% and 14% for V1, V2 and V3 respectively. To investigate the accuracy of the given germination rate, another germination test was performed using 200 seeds randomly taken from each three varieties. Afterwards, one thousand seeds were used from each variety (total of 3000 seeds from the three varieties) to establish the classification model. A detailed flowchart illustrating the seed viability analysis using the near-infrared hyperspectral imaging (NIR-HSI) technique is shown in **Figure 1**.

### HSI system for image acquisition

**Figure 2:**
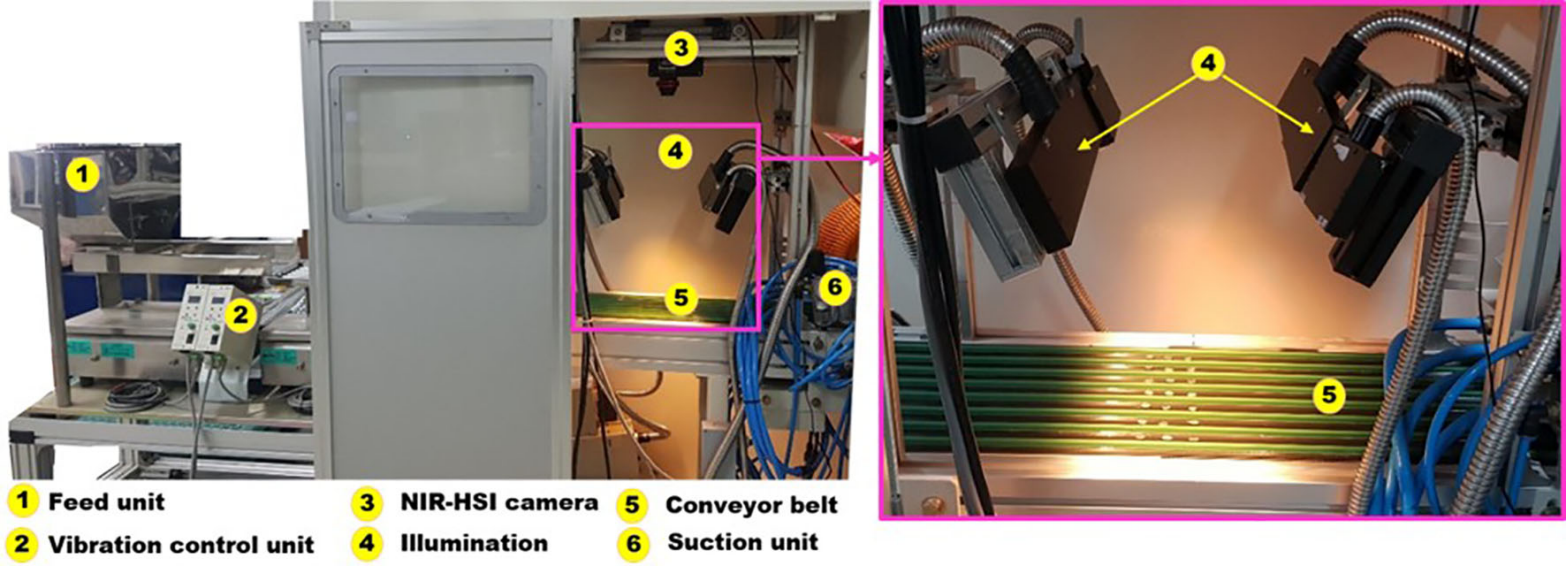
Online NIR hyperspectral imaging system employed for the visualization of seeds in relation to their viability.

In this study, a NIR-HSI camera (Resonon PIKA-640, NIR, MT, USA) with an Allied vision detector combined with 336 pixels and a spectral range of 900–1700 nm was used. Illumination was provided using two pairs of three lighting sources with a tungsten halogen lamp (LS-F100HS, 100 W each) through optical fiber fittings (G(P)L30 × 1.0-1000F). The focal length of the camera was 25 mm, and the distance from the camera lens to the sample was 56.4 cm with an instantaneous field of view (IFOV) of 0.60 milliradians. The conveyor unit was designed with rubber material to prevent seed falling off in the movement process, and the dimensions of the conveyor belt were 200 cm (length) × 18 cm (width), which permitted the continuous analysis of the bulk lot of seeds. The field of view (FOV) of this setting was 26 cm × 18 cm. A DC motor controller was connected to the conveyor belt to control the belt speed using a custom-built algorithm scripted in Matlab (2019a, The MathWorks, Natrick, MA, USA) software. **Figure 2** illustrates the proposed data collection process with a conveyor belt speed of 49 mm/s at an ambient temperature of 21°C.

For model development, to mitigate the prediction error caused by the seed side facing the camera, seed samples were scanned from both sides of the seeds, which doubled the acquired spectra number. The camera exposure time was set to 17 ms during spectral acquisition. Hyperspectral reflectance images of the seed samples were collected and stored for further analysis. Finally, the relative reflectance was calculated using the following equation:


$$X_{cal}=\;\frac{X_{raw}-X_{dark}}{X_{ref}-X_{dark}}$$

where *X_raw_* is the raw HSI image, *X_dark_* is the dark reference acquired by the covered camera lens, *X_ref_* is the white reference obtained using a Teflon board with >99% reflectance, and *X_cal_* is the calibrated image, which was corrected by removing noise to produce a cleaned absolute image (Mo et al., 2014).

### Spectral data processing

The samples containing spatial noise in the collected HSI images were minimized using a 2D median filter with a moving window of 3 × 3 pixels. The regions of interest (ROIs) were used to manually extract the spectral information of the watermelon seed samples from the acquired HSI images. The mean spectral data from each sample were saved according to the respective seeds, and this procedure was performed for all three varieties, resulting in a total of 6000 spectra (1000 seeds from each of the three varieties with double-sided spectra). All seeds and spectra were numbered carefully, and the seeds were placed on a 96-well plate and stored in the refrigerator at 4°C until further analysis.

### Germination test

The between paper germination test is the most effective approach for ensuring easy counting of the sprouted seeds through visualization of the seeds that are beginning to sprout and those that are not showing any action of germination. Due to being naturally aged, the numbers of seeds in the viable and nonviable groups were not the same. The germination rate was found 89% for V1, 32% for V2, and 14% for V3. These rates were determined by performing a germination test on 200 seeds from each variety, and the values were the same as those provided by the seed company. The germination test was done to aid in the classification model development and to inspect the primary germination rate and was conducted following the guidelines of the International Seed Testing Association (ISTA) (International Seed Testing Association, 1985).

Seeds were placed on a moistened paper towel to determine the seed viability after spectral acquisition. The seed number was maintained very carefully from the 96-well plate. All the moistened towels were rolled carefully and placed upright in a deep bottom plastic box. Then, 3 cm of the rolls was covered from the bottom with distilled water and moistened when necessary. The rolled paper towels were placed in the germination cabinet at 25°C for 14 days. Seeds with a primary root length of 5 mm were considered viable. Data from the germination test were used to divide the seeds into two groups: viable seeds and nonviable seeds. After 14 days, the seeds that did not show any signs of germination were included in the group of nonviable or dormant seeds. To remove model bias for any single group (viable or nonviable), the same number of seed samples was included in both of the two groups for each of the three varieties. The details of the sample distribution for the calibration and validation sets for each of three groups are given in **Table 1**.

**Table 1:** Details of the calibration and validation set distribution. The total sample number for each variety is given in parenthesis in the first column.

Variety	Number of samples were taken	Germination rate (%)	Sample number (viable + nonviable)	Calibration samples		Validation samples
V1	1000	89	126		84	42
V2	1000	32	612		408	204
V3	1000	14	242		160	82

### Model development and multivariate analysis

For each of the three varieties, 70% of the data were used to build the classification model and the remaining 30% were applied for the model testing as presented in **Table 1**. Eight different preprocessing methods, including normalization (mean, range, and maximum), multiplicative scatter correction (MSC), standard normal variate (SNV), and Savitzsky–Golay derivatives (first and second order), were applied to the acquired spectral data.

Preprocessing was performed to remove the spectral irregularities that may have been caused by light scattering or the sample texture. Normalization generally compensates for variations the source intensity, resulting in a scaled and offset corrected spectrum at the same time (Lasch, 2012). MSC and SNV preprocessing remove undesired scattering and slope effects (MacDougall et al., 1985; Barnes et al., 1989). The Savitzsky–Golay derivatives represent one of the most popular filters for smoothing spectra by resolving overlapped peaks and removing the baseline offset (Savitzky and Golay, 1964).

For model classification, PLS-DA was used, which is the modified form of the partial least square regression (PLS-R) and is expressed as


Y=X×b+E,

where *X* is an n × p matrix that holds the spectral values of each class, b is the regression coefficient, and E is the error term. In this study, for the construction of the PLS-DA model, the spectral data of viable and nonviable seeds were arranged in matrix *X*, while matrix *Y* contained an artificial value expressing the class, as given below:


$$Y=\{\begin{array}{c} 0=sample\;belongs\;to\;nonviable\;group\\ \;\;\;\;\;1=sample\;belongs\;to\;viable\;group\;\;\;\;\;\;\;\;\;\;\;\;\; \end{array}$$

To classify the samples correctly, a classification threshold value of ±0.5 was generally selected with respect to each group. Samples within the range of ±0.5 from a group were classified as belonging to that group. To build a linear relationship between the predictors and response variables, both *X* and *Y* values were changed by latent variables (LVs):


$$X=TP^{T}+E_{X\;\;}$$

$$Y=UQ^{T}+E_{Y\;\;}$$

Here, *P* and *Q* represent the loading matrix, and *T* and *U* represent the score matrix. *E_X_* and *E_Y_* are the residual matrices of X and Y, respectively.

The goal of this classification model is to detect naturally aged seeds in a nondestructive manner, which may discard some viable seeds with the nonviable group. After applying the final classification model using the NIR-HSI technique, separate dataset (which will be termed as validation set-2 for further) were used to obtain a realistic estimation of the prediction performance. The seed samples of validation set-2 were used to assess chemical differences and analyze the moisture content after detecting by the proposed NIR-HSI technique and performed another germination test.

### Image processing

After applying several preprocessing methods, the model that yielded the best level of accuracy was accepted. To distinguish between viable and nonviable naturally aged seed for each three varieties, the regression coefficient vectors were multiplied by the original masked HSI images to develop the chemical images. The resulting chemical images were converted into binary images using same classification threshold value that was used to construct the classification model to detect seeds in relation with viability. In this classification model, 1 is denoted as viable seeds and 0 for nonviable seeds.

**Figure 3:**
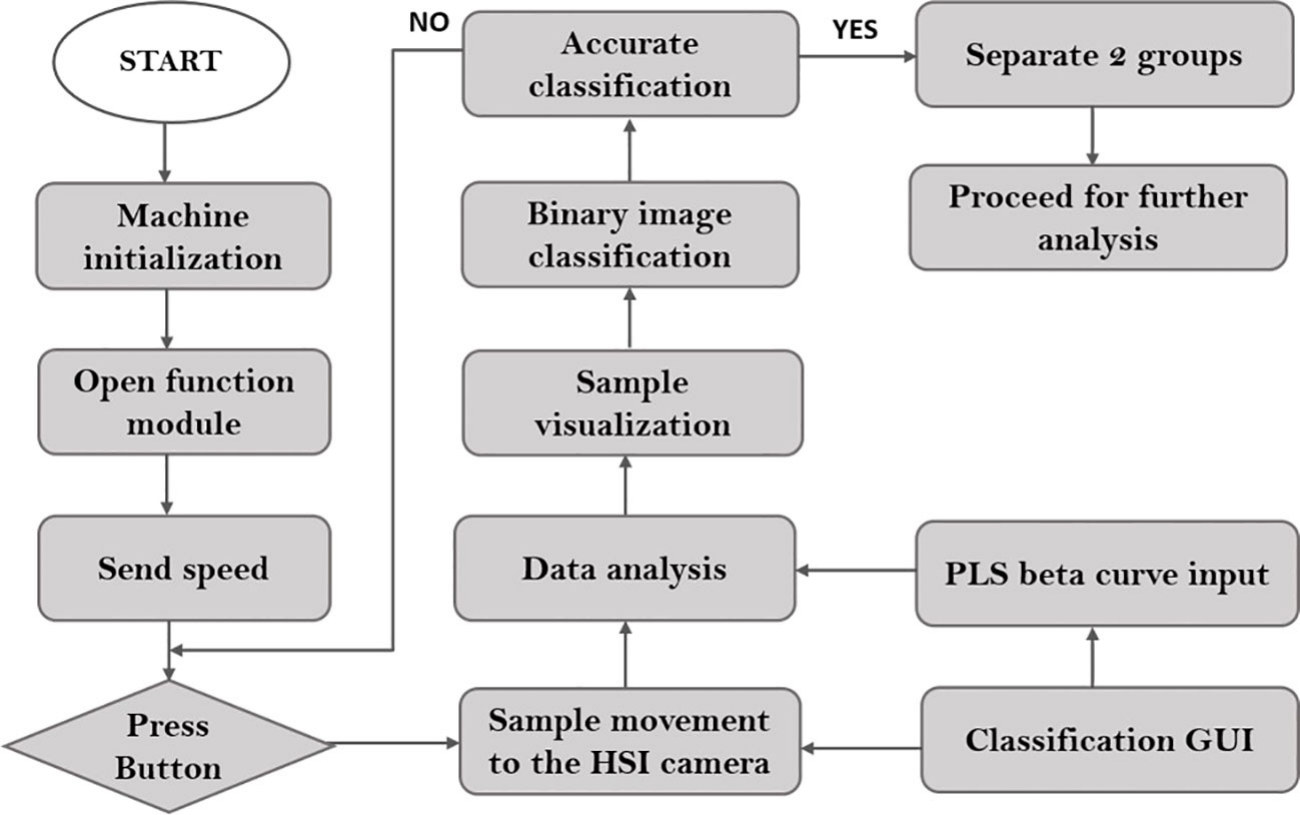
The flowchart of the custom-built algorithm for online measurement.

After model selection, illustrated in **Figure 3**, the proposed NIR-HSI technique was used to inspect the quality of the seeds from validation set-2 as a continuous process. Once the button of the conveyor belt was pressed, samples proceeded to the field of view (FOV) of the NIR-HSI camera. This camera analyzed and identified naturally aged seeds in relation to their viability. After identifying the viable and nonviable seeds from each of the varieties as batch identification, were stored carefully for further analysis.

### High-performance liquid chromatographic analysis

HPLC analysis was carried out on a modular chromatograph containing an evaporative light scattering detector (Alltech 3300 ELSD, HP 1260) and a diode-array detector (DAD) at a wavelength range of 190–800 nm with a quaternary pump. An analytical column C18 (zorbax Eclipse XDB, 150 × 4.6 mm, 50 mm particle size, and 5 µm pore size) was eluted at ambient temperature at a flow rate of 1 mL/min. The injection volume for the liquid samples was 20 µL. Samples were treated differently for carbohydrates and proteins for the HPLC analysis, and the chromatograms were evaluated with an in-built software interface.

Several chromatographic analyses of carbohydrates have been conducted using 190 nm for sugars (Oldfield, 2019), 240 nm for phenylisocyanate (Herrero et al., 2011), 245 nm for saccharides (Zhang et al., 2003; Strydom, 1994), 265 nm for carbohydrates (Yan, 2014), and 280 nm for typical syrup (Kearsley and Dziedzic, 1995). Considering previous studies, several UV wavelengths (nm) were applied, and the chromatograms were recorded to identify the difference in carbohydrate content based on seed viability for each of the three varieties. In total, 30 seeds from validation set-2 (15 viable—5 viable seeds from 3 varieties and 15 nonviable—5 nonviable seeds from 3 varieties) were taken randomly to prepare the sample after detection by the proposed NIR-HSI technique. The seed samples were powdered after removing the seed coat and prepared for HPLC analysis. The procedure used was that described by Lopez-Hernandez et al. (1994) where 1.5 g endosperm was taken and shaken for 30 min with 80 mL of 80% analytical alcohol (Lopez-Hernandez et al., 1994). The extract was filtered, and the residue (alcohol-insoluble solids (AIS)) was reserved for carbohydrate determination. In this study, the endosperm of each individual seed was extracted and shaken for 30 min with 80% analytical alcohol.

Chromatographic analysis of protein has been carried out for various types of agro foods, such as oat (Lapvetelainen et al., 1995), maize (Wilson, 1991), and cocoa seed (Voigt et al., 1994) at 210 nm; soybean (Oomah et al., 1994), whey (Sturaro et al., 2016), milk (Baranyi et al., 1995), and sunflower seed (Kortt and Caldwell, 1990) at 214 nm; milk (Chabance et al., 1995) and pea (Chambers et al., 1992) at 220 nm; lupinus spices (Salmanowicz, 1995), peanut seed (Basha, 1988), and milk (Baranyi et al., 1995) at 280 nm. Based on previous research, in this study, several types of detector (nm) were selected for the HPLC analysis. For sample preparation, another set of 30 seeds (15 viable and 15 nonviable) from the validation set-2 were taken after the detection. The seed coat from each seed sample was removed and ground into a fine powder. One milligram of sample was dissolved in 1 mL of buffer solution (0.1% (v/v) formic acid in water) (Aguilar, 2004). To remove undissolved material, the sample was filtrated through a 0.22 µm filter and used for the HPLC analysis for protein difference between viable and nonviable seeds.

### Moisture content measurement

There are several methods that can be applied for moisture content (MC) measurement. The gravimetric method (Bonner, 1981a) was employed in this study to evaluate the MC retention in seeds in relation with their viability. This method is a standard laboratory technique that can be applied to detect the moisture content difference, and it is widely used in a variety of industries (agriculture, construction, chemical, and food). Seed moisture content retention was detected by following the rules of the Association of Analytical Chemists (AOAC, 1995) (AOAC, 1995). The initial weights of both viable and nonviable seeds were measured in grams using four decimal places. After that, the weighted seeds were dried at 105°C for 24 hours and weighed again. Based on the initial and final weights of the samples, the moisture content was calculated, assuming that all weight loss occurred due to the removal of water. In this study, the moisture content was calculated on the basis of wet weight samples and was calculated as follows (Bonner, 1981b):


$$\%\;MC=\;\frac{(W_{w}-W_{d})\times 100}{W_{w}}$$

where *MC* is the moisture content retention expressed as a percentage (%), *W_w_* is the wet weight of samples, and *W_d_* is the dry weight of samples.

After the execution of chromatographic and MC test of validation set-2 an analysis of variance (ANOVA) test was performed separately for those tests. In the study, only one independent variable was considered. The null hypothesis was that the means of all levels would be equal, and the alternative hypothesis was that the means of one or more levels would be different. If the value of *P* was less than 0.05 (P ≤ 0.05), the null hypothesis was rejected; otherwise, the alternative hypothesis was rejected. The analysis was performed using MATLAB (2019a, MathWorks, Natick, MA, USA).

## Results and discussion

### Spectral acquisition

**Figure 4:**
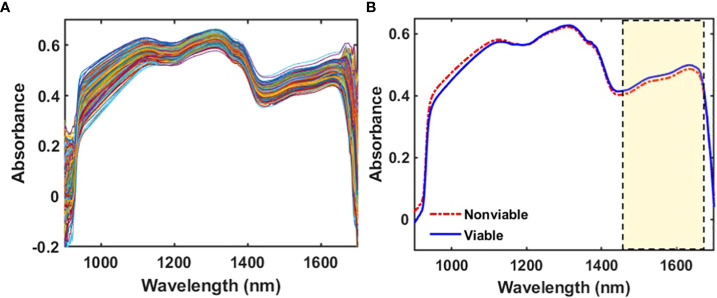
**(A)** Original spectra and **(B)** mean spectra for V3, where the marked region shows a lower intensity for naturally aged nonviable seeds than viable seeds.

After acquiring the spectra of 3000 samples (as both side spectra for the three different varieties were acquired, the total spectral number was 6000), seeds were stored carefully in a 96-well plate by following their respective numbers and varieties. These seed numbers were also used during the germination test, and the seeds were recorded as viable and nonviable for each of the three varieties individually. The original spectra of the seed varieties were extracted from the images using the region between 900 and 1700 nm. Before conducting any kind of analysis on the three varieties, Hoteling’s T^2^ ellipse was used to determine the outliers. However, no outliers were removed as the confidence level was 98.9% for the first two principle components of each naturally aged watermelon seed variety. Finally, the raw and mean spectra (after applying the range normalization as preprocessing) of the viable and nonviable seeds were plotted from the selected ROI and are illustrated in **Figure 4** for V3. Seven different preprocessing methods were applied on the three varieties separately. Among them the best preprocessing method for each variety was chosen based on the prediction accuracy. The mean spectra exhibited a lower intensity for nonviable seeds between 1425 and 1670 nm, which was significantly similar for the other two varieties. This NIR region showed differences for the moisture, carbohydrate, and protein contents (Aenugu et al., 2011). After applying preprocessing methods, the spectra of each of the three varieties displayed major absorbance differences between naturally aged viable and nonviable seeds.

### Model development

Partial least square discriminant analysis (PLS-DA) was performed to establish the classification model. PLS-DA was suitable for use in this study due to the predictors having more variables than the observations and because a high level of correlation was present among the original predictors (Wold et al., 2001). As the predicted values were denoted as 0 (nonviable seed) and 1 (viable seed) in the PLS-DA model, the classification threshold value employed to identify the two groups was automatically set at 0.5, resulting in some misclassified samples in both groups. This phenomenon indicated that some viable seeds had been classified as nonviable and which was inspected later and found very poor seedling growth. Generally, seeds lose their ability to germinate due to aging in storage. Hence, nonviable seeds are always discarded as they are no longer capable of germination (USDA/Agricultural Research Service, 2008). In this study, all the viable seed samples that were present in the nonviable group was rejected. This operation would be helpful to gain a higher germination rate (%) for validation set-2 as the proposed HSI system will only consider the viable seed, which would be beneficial for farmers and seed producing industries.

**Figure 5:**
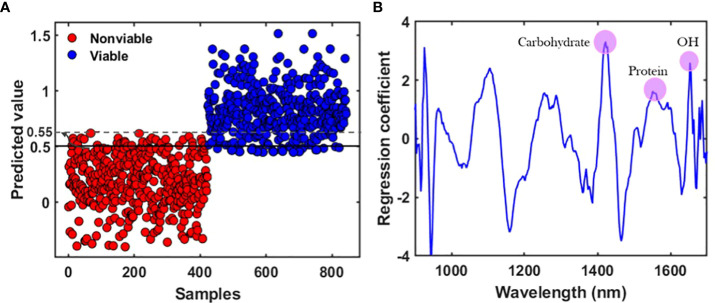
**(A)** Classification result after applying the preprocessing method with a shifted baseline, and **(B)** regression coefficient derived from the partial least square discriminant analysis (PLS-DA) model for the V3 variety.

During the development of the classification model from the prediction set, latent variables (LVs) were calculated in terms of the lower error rate, which is the most common parameter employed to measure the performance of classification models (Ballabio and Consonni, 2013). The classification parameters of the PLS-DA models were collected after applying several preprocessing methods and the only accepted one is presented in **Table 2**, for all three varieties. **Figure 5** shows the classification group and the regression curve for the V3 variety. **Figure 5A** shows some misclassified samples in both the viable and nonviable group. To build a matrix that will contain only the variables of viable seed in that very group, the classification threshold value was shifted carefully. Therefore, it will be helpful to detect viable seeds that will produce healthy seedling from the validation set-2 as the viable seeds that contain very close state to the nonviable group were rejected. Therefore, the classification threshold value was shifted to ±0.53, ± 0.52, ± 0.55 respectively for the three varieties and the similar values will be used for further detection of the validation set-2.

**Table 2:** Calibration and validation set for each three varieties.

Sample name	Viable Seed number	Preprocessing	Threshold	Latent variables (LVs)	Calibration*Acc.v (%)*	Validation*Acc.v (%)*
V1	63	Mean norm	0.53	15	89.0	81.3
		Max norm		14	87.6	79.9
		Range norm		14	88.2	81.1
		MSC		15	82.9	80.0
		**SNV**		**10**	**89.3**	**83.3**
		S-G 1^st^		15	83.7	82.1
		S-G 2^nd^		15	85.7	82.9
V2	306	**Mean norm**	0.52	**8**	**93.4**	**86.7**
		Max norm		10	93.2	84.3
		Range norm		9	90.0	79.9
		MSC		10	92.7	83.5
		SNV		10	91.6	86.1
		S-G 1^st^		9	90.1	85.2
		S-G 2^nd^		9	90.6	85.9
V3	121	Mean norm	0.55	12	94.6	85.6
		Max norm		12	93.7	84.6
		**Range norm**		**10**	**95.6**	**87.8**
		MSC		11	89.9	81.1
		SNV		12	91.2	83.7
		S-G 1^st^		13	92.3	84.8
		S-G 2^nd^		13	93.1	85.2

*Acc.v, Accuracy for viable seed number.

The spectral peak differences for the three varieties were observed from the regression coefficient curve. The regression coefficients were calculated and modeled from the developed partial least square discriminant analysis (PLS-DA) models and used to interpret the different characteristics of seeds. The coefficient curve is the most important factor for multivariate analysis as it gives an interpretation of the results and provides information about wavelength selection (Cheng and Sun, 2015). The coefficients of the PLS model show the highest absolute values of the model, including the most important variables. For the naturally aged triploid watermelon seed V3 variety, spectral peak differences between the viable and the nonviable group were due to the chemical component differences. The regression coefficient curve agreed with the original mean spectra (**Figure 4B**), which also exhibited peaks and valleys at 1415, 1475, and 1605 nm in the NIR region. The NIR region near to 1415 nm is related to C–H bonds, which may be due to the presence of carbohydrates. Additionally, the 1475 nm N–H stretch first overtone may be due to the presence of proteins (Burns and Ciurczak, 2007), and 1605 nm is responsible for the O—H first overtone which may be due to the moisture content (Davies, 2014). The regression coefficient curves for the other two varieties provided similar information.

### Viability prediction and visualization

**Figure 6:**
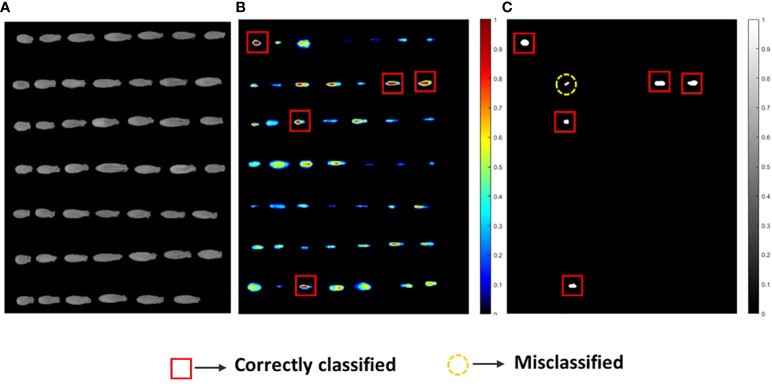
**(A)** The original NIR-HSI images, **(B)** sample visualization using the PLS model where a higher red pixel intensity denotes viable seeds, and **(C)** binary image classification for the V3 seed variety.

After applying the selected preprocessing methods with the classification threshold values, the regression coefficients for each of the three varieties were recorded carefully. Afterwards, the images collected for the seed varieties were evolved by multiplying the clean and masked 3D HSI images with the respective regression coefficients. Post-processing applications, such as masking and morphological area opening on the PLS images, resulted in good classification models. The difference between the two groups in relation to the viability was significantly evident in the middle part of the seed (embryo region), which was predicted by the respective regression coefficient plots. Based on the model accuracy, the classification images were obtained and for V3 the HSI images are illustrated in **Figure 6**.

For clear visualization of the seed viability status, binary classification models of each of the three varieties were developed. Here, for seed detection in relation to the viability, misclassification had occurred for each seed variety as the validation accuracy was near to (≥) 83%. This may have occurred due to the temperature variation in the system, imperfect lighting, and vibration during data collection. Another reason may be due to the dormancy state of the seeds which could be germinated under favorable conditions (Bradford and Nonogaki, 2007). However, the image-based germination accuracy for the prediction group was still quite satisfactory in comparison with the actual germination rate.

After seed detection related to the viability of each variety, seed samples from validation set-2 were used to predict the germination test and the differences in chemical and moisture contents as predicted from the regression coefficients. The PLS models for the three varieties exhibited carbohydrate, protein, and moisture related differences in relation to the viability (**Figure 5B**); hence, these three components were inspected for further analysis. For this purpose, seed samples were placed over the conveyor belt irrespective of sides, facing to the camera, and the speed was similar (49 mm/s) to that used in the model development process. After detection seeds were stored carefully as two groups: viable and nonviable.

### Real-time germination test

The objective of this study was to detect the germination rate (%) of naturally aged seed varieties in a nondestructive way. To investigate this, 590 seed samples of three varieties from validation set-2 were chosen randomly. After classifying seeds into two groups in relation to their viability, a between paper germination test was performed following the similar method that was used to predict the classification models. Here also a 5 mm root length was denoted as viable seed following the International Seed Testing Association (ISTA) rules of viability detection (International Seed Testing Association, 1985). For the viable group, in each of the varieties, the germination rate was moderately increased with healthy seedling emergence. This may happen as the classification threshold value discarded the seeds those were very near to the nonviable group. For the nonviable group, the seed germination rate (%) was lower than (<) 30% for each of the three varieties where the healthy seedlings were less than (<) 7%. **Table 3** presents the final germination accuracy, which indicates an increased germination rate (%) compared to the germination percent provided by the seed company and this was found to be correct by performing another lab-based germination test mentioned in section 2.4. (Germination test) using 200 seed samples from the three varieties. The classification parameters that are presented in **Table 3** as ‘Final germination rate’, proved the efficiency of the classification models developed by the proposed online NIR-HSI system.

**Table 3:** The germination rate detection accuracy from validation set-2 and its confusion matrices.

Variety	Preprocessing	Given germination rate (%)	Final germination rate (%)	Confusion Matrices
V1	SNV	89	91.4	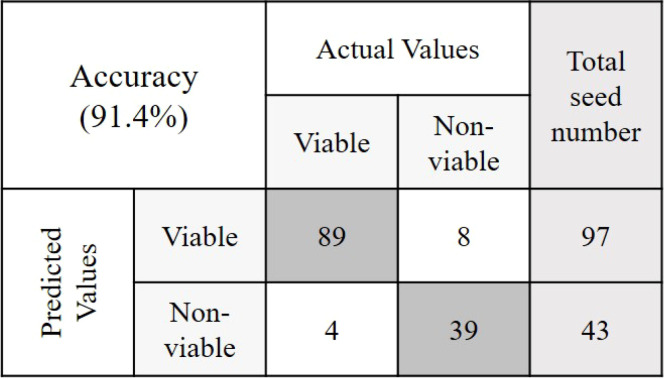
V2	Mean normalization	32	80.5	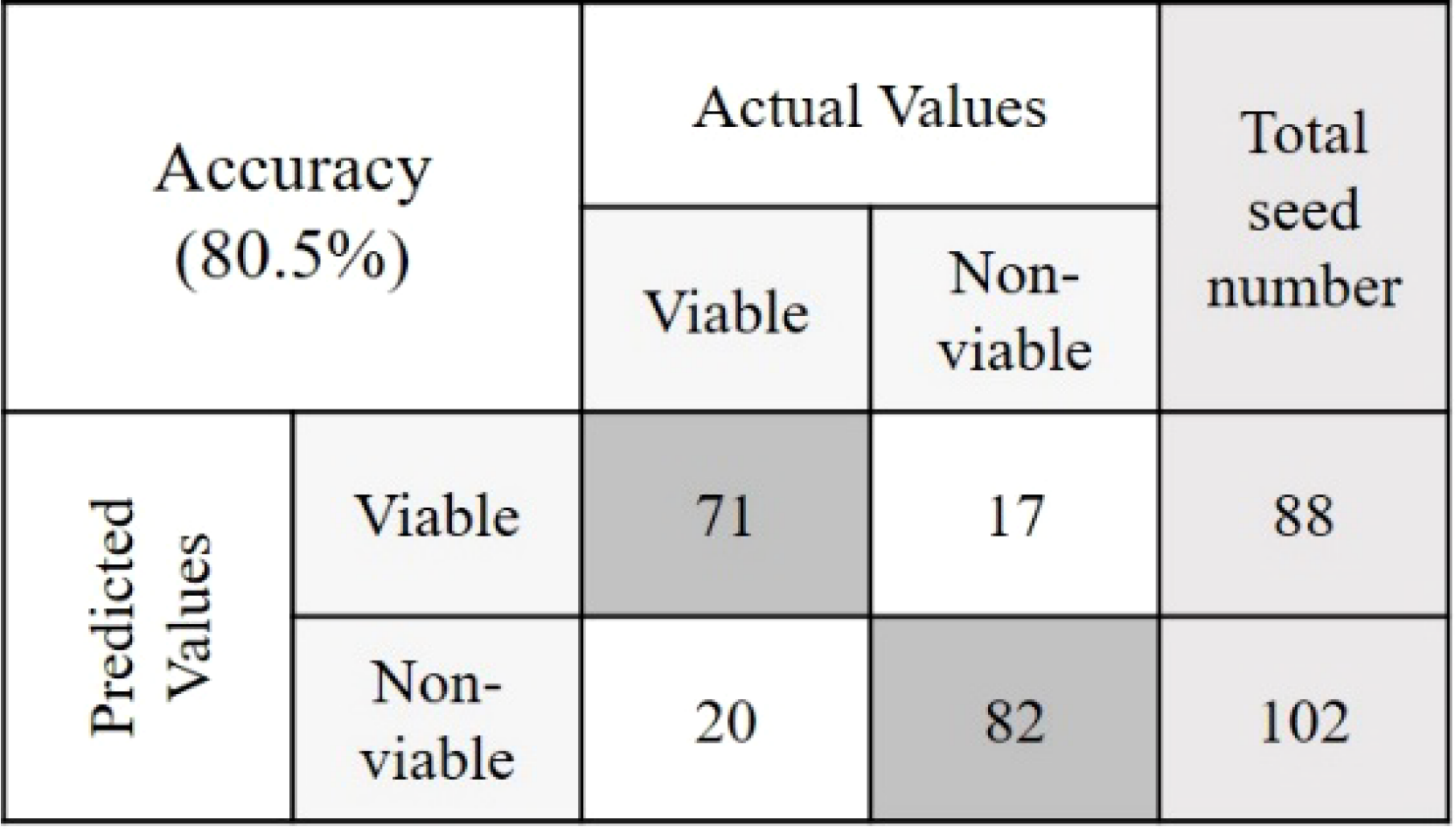
V3	Range normalization	14	77.7	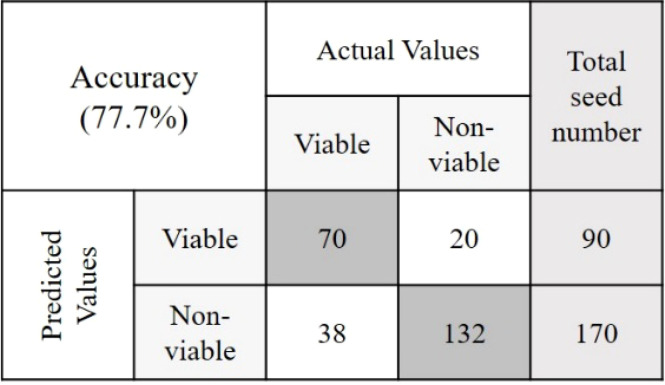

### Chromatographic analysis

To evaluate the chemical difference in viable and nonviable seed, chromatographic analysis was carried out after the detection of seeds in relation to viability. Seed samples were taken randomly of each three varieties from the validation set-2 as two separate groups (viable and nonviable) from each three varieties. The regression coefficients of the classification models were showing chemical differences between viable and nonviable seed which was consistent for each three varieties. For this analysis, 120 samples in two groups (60 for carbohydrates and 60 for proteins) from all three varieties were taken randomly. In this analysis, the quantities of the denoted chemicals that passed through the detector during chromatographic measurement were compared for viable and nonviable seeds. Here it showed the denoted chemicals contain strong absorption (higher peak area (area %)) for viable seeds and lower for nonviable seeds. Which proves that viable seeds contain a higher amount of carbohydrates and proteins than nonviable seeds. For the three varieties, the detector wavelength was consistent. For carbohydrates and protein detection, the detector wavelength was set to 265 and 220 nm respectively.

**Figure 7:**
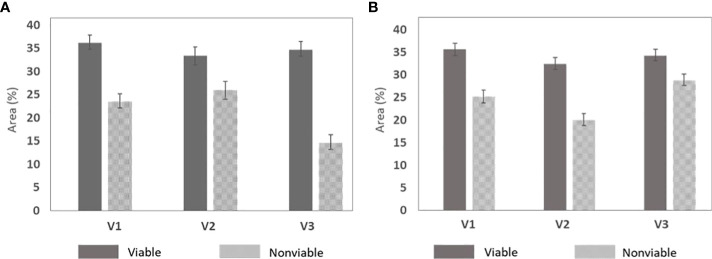
Chemical difference between the naturally aged viable and nonviable group that was developed by taking five random seeds from the three varieties. **(A)** Carbohydrates and **(B)** protein.

**Table 4** shows the chemical differences between the two groups of naturally aged triploid seeds in relation to their viability. Each of the varieties exhibited a steady result for the two groups. A graphical representation of the variability of the data was used, as shown by the error graph in **Figure 7**, demonstrating that the carbohydrate and protein differences were statistically significant in relation to the viability for each variety of naturally aged watermelon seed.

**Table 4:** Reverse-phase high-performance liquid chromatography (RP-HPLC) test results showing the chemical composition differences.

Variety	Number of samples	Chemical component	Peak signal (nm)	viability	Retention time	Area %
V1	20	Carbohydrates	265	viable	35.163	**36.1586**
				nonviable	35.163	23.4867
	20	Protein	220	viable	35.246	**35.6213**
				nonviable	35.246	25.1953
V2	20	Carbohydrates	265	viable	35.242	**33.3985**
				nonviable	35.242	25.9633
	20	Protein	220	viable	35.034	**32.4334**
				nonviable	35.034	20.0022
V3	20	Carbohydrates	265	viable	35.035	**34.6988**
				nonviable	35.035	14.5625
	20	Protein	220	viable	35.156	**34.1842**
				nonviable	35.156	28.6951

### Moisture content measurement analysis

**Figure 8:**
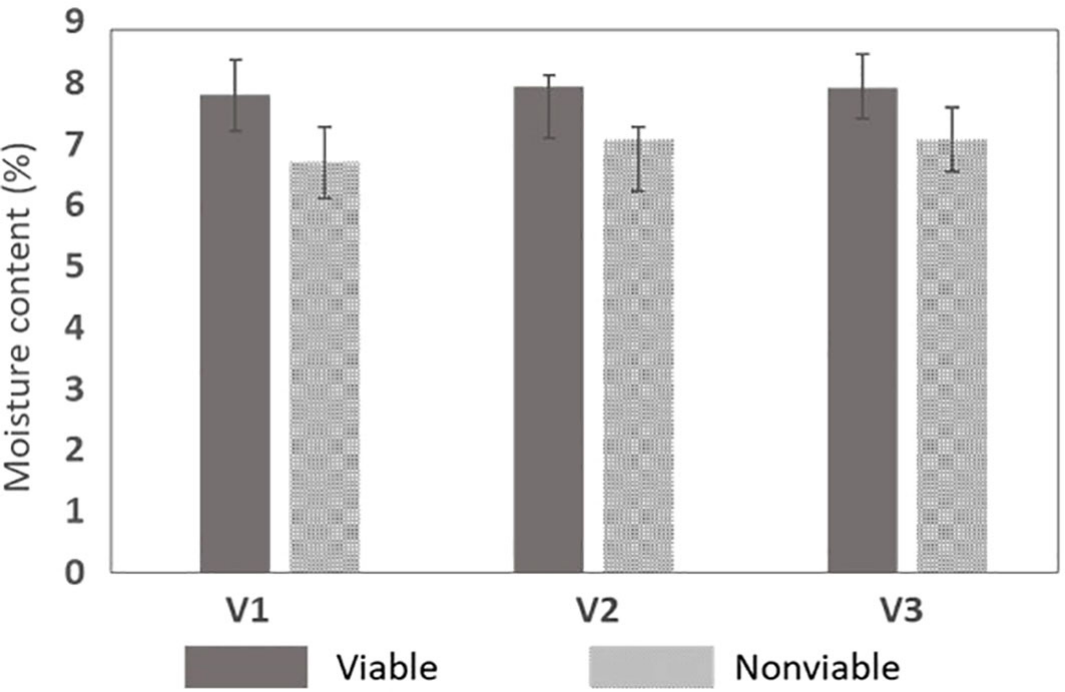
MC retention test between the naturally aged viable and nonviable seed for the three varieties.

The targeted MC retention for naturally aged seeds in relation to the viability displayed similar trends to those shown in the chromatographic analysis. For this analysis, a total of 120 seeds (60 viable and 60 nonviable seeds from all varieties included in validation set-2) were used and a one-way analysis of variance (ANOVA) was performed to evaluate the performance. As shown from the test results in **Table 5**, a significant difference (P< 0.05) existed between the groups (viable and nonviable seeds). Another graphical representation of the variability of the MC data shown by the error graph in **Figure 8** represents that the MC in nonviable seeds was less than that of viable seeds.

**Table 5:** Statistical parameter difference for moisture retention between viable and nonviable seeds.

Variety	Number of samples	Mean square	*F* value	Prob > *F*
V1	40	3.189	14.39	0.001*
V2	40	3.993	12.98	0.002*
V3	40	4.182	14.02	0.001*

*Significantly different.

### Comparison with deep learning techniques

Deep learning techniques have recently emerged as a promising tool for replacing conventional machine learning classifiers due to better classification performance and the lack of redundancy of the feature extraction from handcrafted images (Krizhevsky et al., 2012; Cireşan et al., 2013). Deep learning techniques can autonomously extract image features based on artificial neural networks, performing high-accuracy detection and classifications. In the agricultural field, deep learning networks such as a convolutional neural network (CNN), AlexNet, VGG-19, and residual network (ResNet) have been recently used for morphological pattern classification of watermelon seeds (Ahmed et al., 2020), detection of seedless from seeded watermelon seeds (Mukasa et al., 2022), and automatic detection of chickpea varieties (Taheri-Garavand et al., 2021).

However, despite its high accuracy, deep learning techniques have hardly been applied to the optical sorting system because of their relatively heavier computation load and cost than machine-learning methods (Heo et al., 2018). In our previous study, the cost of deep-learning based sorting system is mainly affected by the computational cost (US$60000), including high-quality central processor units, graphics cards, and multiple NIR cameras (each US$15000) (Mukasa et al., 2022). A cost-effective real-time recognition system for detecting full-surface defects of soybean has been recently developed using various CNN models (Zhao et al., 2021). However, its total cost was not revealed to readers. Therefore, in the current study, the PLS-DA model, a machine-learning-based classifier, was suggested for real-time online measurement of the viability detection of naturally aged watermelon seeds.

### Impact on genetic resources

Detecting the seed viability is essential for maintaining seed germplasm conservation. Most germplasm collections for the *Cucurbitaceae* crop family are provided by the Cucurbit Genetics Cooperative and the United States Department of Agriculture (USDA) for breeders and entrepreneurs interested in the genetic information and breeding of species. In the USDA germplasm collections, some of the seeds (approximately 10%) are used to increase seed germination and quantity. Furthermore, the breeding program for watermelon cultivars is usually performed by intercrossing the best cultivars which are currently available or crossing the outstanding cultivars with accessions including more valuable features missing from the outstanding cultivars (Whitfield and Last, 2017). Accordingly, it is important to acquire seeds of the best vigor cultivars, a set of accessions from germplasm resources having high-quality and valuable genes.

## Conclusion

In the current study, NIR-HSI system and PLS-DA classification models were developed for the viability detection of naturally aged watermelon seeds. The classification models employed in this study were focused on the detection of viable seeds, which rendered some viable seeds as nonviable. Based on the developed PLS-DA model outcome, resultant images for viability visualization of watermelon seeds were acquired with the validation accuracy of 83%. The image-based accuracy was relatively higher than the actual germination results. Finally, the potential of the online measurement of the developed NIR-HSI system has been demonstrated through a continuous inspection process by a custom-built scripted algorithm. The chromatographic and MC retention tests on the inspected viable and nonviable seed groups showed significant differences in carbohydrate, protein, and moisture contents.

Deep-learning-based optical techniques can be another option for agricultural sorting systems due to their high accuracy. However, its total cost and computational load are still much higher and heavier than the machine-learning-based system. Moreover, developing a real-time online sorting system will be beneficial in maintaining seed germplasm conservation and conducting the breeding program. Therefore, in this study, the PLS-DA-based VIS-NIR system showed high potential for real-time online measurement of the viability detection of naturally aged watermelon seeds. Using the developed system, seeds can be inspected rapidly, which benefits farmers, breeders, and seed companies. In our future work, we will develop an automated online seed sorter integrated with the developed VIS-NIR HSI system for watermelon viability sorting.

## Data availability statement

The original contributions presented in the study are included in the article. Further inquiries can be directed to the corresponding authors.

## Author contributions

JY and MA conceived the overall contents and structure for this article. JY and B-KC led the data analysis drafted tables and figures. SL, PM, JK, and HL analyzed sample information and conducted experiment. GK and B-KC reviewed successive drafts. All authors contributed to the article and approved the submitted version.

## Funding

This research was supported by Chungnam National University, Republic of Korea.

## Conflict of interest

The authors declare that the research was conducted in the absence of any commercial or financial relationships that could be construed as a potential conflict of interest.

## Publisher’s note

All claims expressed in this article are solely those of the authors and do not necessarily represent those of their affiliated organizations, or those of the publisher, the editors and the reviewers. Any product that may be evaluated in this article, or claim that may be made by its manufacturer, is not guaranteed or endorsed by the publisher.
